# 

**Published:** 1878-05

**Authors:** 


### KENTUCKY STATE DENTAL SOCIETY.

The annual meeting of this body will be held in Covington, Tuesday, June 4th, 1878, when it is expected a large number of the profession will be present, and a very interesting and profitable time is anticipated.

The programme here given, presents a large range of subjects, in all of which the profession is deeply interested. Let every one come with preparation to add something of interest to the occasion.

Do not let all the work devolve on the committees. They are as follows with the subjects of each:

ANATOMY.

Do the nerves of extracted teeth unite in replanting when in a healthy condition? What are the condition of the alveolar processes when there is abscess on the tooth? Drs. S. Driggs, Wm. Rogers.

PHYSIOLOGY.

What are the provisions of nature for the protection of exposed pulps? Circulation of the pulp vessels. Drs. Wm. H. Goddard, J. Hooper.

PATHOLOGY.

Secondary dentine; osseous pulp; abnormal secretions; exostosis; action and effects of dead pulps. Drs. A. O. Rawls, Wm. VanAntwerp.

CHEMISTRY.

The different agents that act to produce decay, or disintegration of dentine and enamel and their mode of action. Prof. J. S. Cassidy, Dr. G. E. Dunn.

HYGIENE.

General systemic treatment as a prophylactic. Use of phosphates and the results. Care of the teeth in general. Drs. Thos. H. N. Smith, W. W. Justice.

THERAPEUTICS.

Particular treatment of neuralgia. Action of therapeutic agents. Drs. W. G. Redman, Jno. T. McMillan.

SURGERY.

Surgical operations on the pulp. Best means of extracting pulps. Replanting teeth. Transplanting teeth. Drs. F. Peabody, C. G. Edwards.

ANÆSTHETICS.

Uses and abuses of anæsthetic agents. The principles of their action. Local and general agents. Drs. B. O. Doyle, J. F. Canine.

VOLUNTARY ESSAYS.

Treatment of children's teeth. Drs. A. Wilks Smith, Wm. Wasson.

OPERATIVE DENTISTRY

With clinics, by Prof. H. M. Reid and W. S. Moores.

EXECUTIVE COMMITTEE.

W. H. Goddard, W. W. Justice, J. T. McMillan.

President, A. W. Smith, Richmond; Vice-President, C. G. Edwards, Louisville; Secretary F. Peabody, Louisville; Treasurer, J. F. Canine, Louisville.

BOARD OF CENSORS.

S. Driggs, Lexington; F. Peabody, J. Hooper.

Let the profession outside the state be well represented.

### IOWA STATE DENTAL SOCIETY.

Theenth Annual Meeting of the Iowa State Dental Society will be held at Iowa City, commencing on the ninth day of July, 1878, and continue three days.

ORDER OF BUSINESS.—FIRST DAY.

At ten o'clock a. m., the members will meet at the lecture room of the medical department of the State University for general greeting and payment of dues.

At half past eleven o'clock a. m., an address of welcome will be delivered by Prof. Clapp, of Iowa City, responded by Dr. Ingersoll, of Keokuk.

At two o'clock p. m., the following regular order of business will be taken up.

Organization and roll called.

Application for membership and election of members.

Report of executive committee.

Unfinished business.

Selection of place for next meeting.

Election of officers.

Retiring president's address and introduction of new officers.

At evening session, eight o'clock, the question of permanently locating the society will be discussed and voted upon.

SECOND DAY.

The morning session will be taken up in clinics and microscopical observation.

The afternoon session will be devoted to the reading and discussion of essays.

THE THIRD DAY

Will be devoted to the reading and discussion of essays, and the transaction of such other business as may come before the society.

The essayists who were elected last year will read papers upon the following

SUBJECTS.

Diagnosis. By Dr. L. C. Ingersoll, Keokuk.

Where is the Fault? By Dr. R. L. Cochran, Burlington.

Why do our Fillings Fail? By Dr. I. D. Pearce, Iowa City, and E. J. Woodbury, Council Bluffs.

Materials for Filling Teeth. By Dr. I. P. Wilson, Burlington.

Preservation vs. Devitalization of the Dental Pulp. By Dr. I. B. Morgan, Davenport.

Therapeutics. By Dr. F. W. Dean, Des Moines.

In addition to the above, there will be volunteer papers from eminent scientific men from other societies.

CLINICS

Will be conducted by the following gentlemen:

Dr. L. C. Ingersoll, Keokuk; Dr. O. H. Denise, Burlington; Dr. G. W. Fuller, Des Moines; Dr. E. J. Woodbury, Council Bluffs; Dr. T. K. Brewster, Oskaloosa; Dr. W. P. Dickenson, Dubuque.

Dr. S. A. Garber, of Tipton, will demonstrate a gold plate.

### CONTENTS.

### COMMUNICATIONS.

### *THE DENTAL REGISTER,*

A MONTHLY MAGAZINE DEVOTED TO THE INTERESTS OF

### THE DENTAL PROFESSION.

***Terms Reduced to $2 50 per Year. Single Copies 25c.***

### *EDITED BY PROF. J. TAFT, D. D. S.,*

AND PUBLISHED BY

SPENCER & CROCKER, Ohio Dental Depot.

The volume commences in January and closes in December.

The REGISTER being issued monthly is always fresh, therefore Indispensable to the practitioner who desires to keep posted up with the new inventions and improvements which are daily developed. Neither expense nor effort will be spared to give value and variety to its contents. Articles will be illustrated with well executed engravings whenever they will add to the interest or assist to explanation.

Its extensive circulation and frequency of issue make it one of the BEST DENTAL ADVERTISING MEDIUMS in the United States.

All subscrptions must begin with January or July.

Local or special notices, five lines of less, will be inserted for $3.00 each insertion; over five lines, 50 cts. per line.

All advertising bills payable in advance, or at furthest, after the issue of the first number containing the advertisement. All transient advertisements to be paid for in advance.

☞ CONTRIBUTIONS SOLICITED.

Exclusively Editorial Communications should be addressed to Editor.

The latest number of the REGISTER may be ordered with or without other goods at any time, and all letters should be addressed to

**SPENCER & CROCKER, Ohio Dental Depot, Cincinnati, O.**

